# Supplementary material for: Patterns of Intron Gain and Loss in Fungi
Source: PLoS Biol. 2004 Nov 30;2(12):e422. doi: 10.1371/journal.pbio.0020422 (PMC532390; doi:10.1371/journal.pbio.0020422)
Supplement: Table S1 — Also available at http://genes.mit.edu/NielsenEtAl/. (4.3 MB ZIP). [file pbio.0020422.st001.zip › NielsenEtAl/html/322.html]

AN4304.1.NCU04098.1.MG09542.1.FG04567.1


```
 CLUSTAL W (1.82) Multiple Sequence Alignments - Introns Inserted


Sequence 1: AN4304.1	149 aa
Sequence 2: FG04567.1	159 aa
Sequence 3: NCU04098.1	165 aa
Sequence 4: MG09542.1	167 aa
Alignment Length: 170 aa
Number Identitical Residues: 79 aa
Alignment Score (without introns) 3595


MG09542.1 	MISSRFMG~G0NILR~QASRTACRAPAHSIAAPTLLRQLQAQQQRRLLTSEVRQAIDKAV
NCU04098.1	MLTRSLFS~R0QLFA~AASRPAIAPKAVSSAFRPVL-----FYQNRFLSDATRQAIDKAV
FG04567.1 	MFARTVAS0S~--LR~QAARPMAVR-STASAFRTPIAPL-SPFQIRLLSDQTRSAIDKAV
AN4304.1  	MFSRTAIS~-~---F0TLRRSLFRP-QLSPQFPNQLP---SVLQARLLSTETKAAIDKAV
          	*::    .           *.       :      :    :  * *:*:  .: ******

MG09542.1 	ASAPVVLFMKGTPETPQCGFSRASIQILSMQGVDPEKFAAYNVLEDQSLREG~IKEYSEW
NCU04098.1	ASAPVVLFMKGTPETPQCGFSRASIQVLGLQGVDPNKFAAFNVLEDAELRQG1IKEYSDW
FG04567.1 	SSAPVVLFMKGTPETPQCGFSRAAIQILGLQGVNPDKFAAFNVLEDPELREG~IKEYSDW
AN4304.1  	ASAPVVLFMKGTPETPQCGFSRASIQILGLQGVDPKKFVAFNVLEDPELRQG1IKEYSDW
          	:**********************:**:*.:***:*.**.*:***** .**:* *****:*

MG09542.1 	PTIPQVYIDKEFVGGCDILVSMHQSGELGKMLAEKGVLVKGQEGEADNKSE---
NCU04098.1	PTIPQLYIDKEFVGGCDIIVSMHQNGELAKLLEEKDVLVKGEEGAAEEQTEKKE
FG04567.1 	PTIPQLYVEKDFIGGTDILVSMHQNGDLAKLFDEKKVILEGEPEQKE-------
AN4304.1  	PTIPQLYLNKEFIGGCDILMSMHQNGELSKLLEEKGVLVAE-------------
          	*****:*::*:*:** **::****.*:*.*:: ** *::
```
